# On Some Points Connected with Cholera

**Published:** 1889-08-24

**Authors:** 


### On Some Points Connected with Cholera.

*(Continued from page 306.)*

### THEORIES EXAMINED.

Next we have cholera attributed to lunar influences, to epidemic constitutions of the air, to a *constitution medical cholerique*, all being more or less relics of the ideas which in the Middle Ages attributed epidemics to comets, earthquakes, volcanoes, and sidereal changes, magnetism or electricity taking the place of Saturn or Mercury in the scheme of causation. Extraordinary meteorological changes have been much favoured as a cause, and doubtless previous to or during some epidemics extraordinary meteorological conditions have been observed. Thus Sir Guyer Hunter states that previous to the Egyptian epidemic of 1883 there was a still, cloudless atmosphere, saturated air, and a peculiar yellow haze called by the Arabs "el hawa el asfar," and it was noticed that sparrows deserted the locality where the haze appeared. Dr. Kirke's Egyptian meteorological records also show that in June, 1883, in Egypt, as compared with previous times, the mean barometric pressure was somewhat below and the temperature somewhat above the average. The maximum relative humidity was also highest, while ozone and wind force were low. From the 12th to the 20th of June, cholera appearing on the 15th, there was a period of excessive relative humidity, with occasional stagnancy of air. In the Crimea, the outbreak of disease was coincident with change from dry to humid atmosphere; at Agra, in 1884, with a dry east wind. Others have associated cholera with fogs and mists. Bellew, in his recent "History of Cholera in India from 1862 to 1881," regards it as an intestinal catarrh produced by atmospheric conditions, so strictly analogous to epidemic catarrh or influenza that the etiolgy of one applies to the other." Still no special meteorological phenomenon can be regarded as the cause of, or essential to, cholera; for while peculiar atmospheric conditions have frequently been noticed, cholera has often occurred when no such conditions were noted, although meteorological observations were conducted on the spot. On the other hand, such conditions have prevailed without cholera. The fact appears to be that great heat, wide ranges of temperature, disturbances of atmospheric electricity, great magnetic disturbances (usually present when the largest sun spots are seen), abnormal falls of rain, are all more or less predisposing agencies, although none are decidedly necessary as existing causes.

Next, we have cholera attributed to malarious influences, a theory which has obtained many advocates. Under this head is worth recalling the Sanskrit term for cholera — "vishuchuka," which means fever and incessant purging, thus coupling the two maladies together. McCulloch, so far back as 1827, wrote: "Cholera belongs, predominarilly, if not exclusively, to the same climates, the same soils, or generally to all those countries in which other diseases of malaria abound;" and afterwards he remarks that the disease is common where fevers are common, and rare where fevers are rare. Fayrer says an attack beginning with cholera may end as fever, and *vice versâ*. Munro calls cholera and fever one and the same disease. Macpherson states pernicious fever always resembles cholera. Curron, Bellew, Green, and several others have noted the identity of Peshawur fever with cholera. Prof. Blanc, of Bombay, styles cholera "malarious cholera." Macnamara writes the epidemics of Northern India show how constantly those of cholera and malarious fever are associated, and tends to identity of cause. Chevers said several forms of fever have the appearance of forming a link between malarious remittent and cholera. Dr. Smith, A.M.D., wrote a book tending to prove that cholera and fever are identical. On the other hand, Surgeon-General Murray, who saw as much cholera as most people, wrote: "Many of the cases assume an intermittent type, and this appears to be the principal feature of similarity. The algidity of fever which shows itself insiduously in the middle of an attack of intermittent is not the algidity of cholera." There is no difficulty in diagnosing an ordinary case of ague from an ordinary case of cholera, but there may be a difficulty in deciding when, as sometimes occurs, diarrhœa takes the place of the cold fit of ague; and when this occurs we can only wait for further development, whether the hot stage of fever or the rice-water stools and cramps of cholera. Cholera rarely begins with rigors, but it does so sometimes. In an obscure case, the fact or otherwise of cholera being in the neighbourhood would tend towards deciding the question. In temperate climates, where malarious fevers do not occur with the same intensity as in the tropics, such difficulties of diagnosis rarely arise, but in the tropics it is very different.

Next, there is the fœcal theory, early conceived and very generally accepted. Under this idea cholera dejections were regarded as poisonous if introduced into the system. But direct experiments have produced negative results. It therefore became necessary to modify conclusions, and this was done by the theory of some change in cholera evacuations taking place outside the body. C. Macnamara relates an instance strongly corroborative. Some rice-water evacuations were mixed with water and stood exposed to the sun for some hours. Accidentally, nineteen persons drank of this, and five were attacked with cholera within thirty-six hours.

The theory vitalising the poison outside the body necessitated the search for some nidus in which it might develop. This was forthcoming in Pettenkofer ground-water theory, which postulates the soil with being the laboratory. This, however, is a mere elaboration of Pringle's idea, who, in his work on the "Diseases of the Army in Flanders," 1780, stated: "In Dutch Brabant the people are more or less subject to intermittent in proportion to the distance of the water from the surface, so that by looking into their wells one may form a judgment of the comparative healthiness of villages." Pettenkofer's theory has, however, exercised great influence on the minds of many medical men. He takes for granted there is a cholera poison, which, on reaching the soil, undergoes changes which depend on the presence of a certain amount of moisture and of organic matter. But even if this condition be fulfilled, there will be no epidemic unless certain unknown meteorological conditions are present, and a predisposition to the disease in those coming within the influence of the infected area. And Pettenkofer endeavoured to show that cholera is worse in India in accordance with the lowest level of the ground water. But Pettenkofer goes too far when he maintains that a certain condition of ground water is necessary for cholera. Cholera is met with in the semi-desert districts of Western India, where water is 800 feet from the surface (of which districts Pettenkofer knows nothing), and in Bombay and Bengal, where the water is a foot or two from the surface.

The fungoid theory has had many supporters since Grove, Swayne, and Britten, in 1849, published drawings of cholera cells (afterwards proved by Buck to be spores introduced with the food), up to 1883-84, when Koch discovered his cholera comma bacillus. But those who have professed fungi or microbes as the cause are probably mistaken. Lewis and Cunningham, in India, in 1874, proved the non-existence of any specific cell or other body peculiar to cholera, and the remark is applicable to Koch's bacillus. Drs. Klein and Gibbs, who were sent out to India in 1884, refuted Koch's conclusions, and a committee of eminent medical authorities, appointed by the Secretary of State to enquire into the conflicting statements, reported that comma-shaped bacilli are ordinarily present in cholera dejections—also in different parts of the alimentary canal in health—ending with the statement that there are no real grounds for assuming they produce the disease in man. Pettenkofer also, while recognising Koch's bacillus, denies the conclusion that cholera is thereby caused. So did Vandyke Carter, the Bombay microscopist. Another theory is that of Chapman, who attributes cholera to a simultaneous and abnormal superabundance of blood in, and excessive preternatural activity of, the spinal cord and sympathetic nervous system, brought on by heat. The most recent theory is that which attributes the disease to a poison formed within the body.

But notwithstanding all research, argument, and theory, the fact remains that we do not know what is the cause of cholera. All that can be said is, that the present state of our knowledge tends to the belief that there is a something acting primarily on the nervous system—whether it should be called germ, microbe, poison, bacteria, or any other name, we do not know.

If the proposition that a something exists be admitted, the mode of origin and dissemination are the next points. Numerous instances might be cited of cholera presenting in widely different localities under the impossibility of direct intercourse with cholera-stricken persons. The cholera must therefore have been conveyed through the atmosphere, or it must have been the revival of the seeds remaining from a previous epidemic, or it must have originated *de novo*. We confess that we see no reason why cholera poison should not originate *de novo*. There is nothing inconsistent in the admission that what has originated under certain conditions may originate again under the same conditions. Unless one particle of matter is acted on by another particle of matter the result is *nil*. Oxygen and hydrogen placed in juxta-position at an ordinary temperature will not combine; flame being applied, the result is water. Light or heat may be applied, in any amount, to an egg or seed or a dry rotifer, but they will not change if air is withheld from the first, or moisture from the latter. Given certain but at present unknown conditions of matter, certain but unknown conditions of atmosphere, and certain conditions of human constitution, and we fail to see why a disease poison should not be produced, as we know the poison must have been once elaborated.

It was stated above that numerous instances could be cited where the disease occurred under the impossibility of direct intercourse. On the other hand, many other instances might be brought forward where the conveyance of the disease by direct intercourse appeared certain. The fact of people going from one cholera house or from one cholera village to another healthy house or healthy village, their arrival being immediately followed by cholera in themselves and others, cannot be ignored. Especially when it i considered that it is not those who come from any one locality who apparently spread cholera, but those who come from a cholera locality into one not yet infected. So far as we are aware, there are no instances of persons coming from an uninfected locality into another uninfected locality being seized with cholera immediately afterwards, and apparently spreading the disease.

Various observers have adhered to the theory that cholera cannot be carried from an area in which the disease is present to another in which it has not made its appearance in the natural course of seasonable development. But all admit this endemic area is not well defined, but shades off indefinitely. This endemic area has been limited to an absurdly small extent as to part of a ship; the proof being the fact of cholera on board among troops not spreading to the crew. On the other hand, the endemic area has been extended to any part of India or the world as the disease spread there. We do not believe in this endemic area. We do not think there is any place where cholera may not spread, or any place in temperate or tropical climates where it may not originate. That some localities and climates are more favourable to spread than others may be admitted. But the endemic area, according to the inventors of the term, is wherever cholera does spread.

Those who advocate the theory of cholera originating in Bengal, and spreading thence from east to west, are accustomed to call instances when this does not occur "little facts" at variance with the general history of cholera, and to regard them as the exceptions proving the rule. But little facts are as stubborn as great facts.

(*To be continued.*)

